# Outcomes in heart failure patients with third-degree heart block during COVID-19

**DOI:** 10.3389/fcvm.2026.1701620

**Published:** 2026-03-03

**Authors:** Benjamin Kash, Ethan Wahle, Eli Blaney, Saif Zurob, Amjad Kabach, Ali Bin Abdul Jabbar

**Affiliations:** 1 Creighton University School of Medicine, Omaha, NE, United States; 2 Department of Internal Medicine, Creighton University School of Medicine, Omaha, NE, United States; 3 Department of Cardiology, Creighton University School of Medicine, Omaha, NE, United States

**Keywords:** COVID-19 pandemic, heart failure, in-hospital mortality, length of stay, third-degree heart block, total hospital charges

## Abstract

**Objective:**

Heart failure is a common sequela of third-degree heart block. This study examines trends in mortality and utilization of medical resources before and during the COVID-19 pandemic in patients with heart failure and third-degree heart block. We also seek to investigate outcomes for different patient demographics, hospital characteristics, and related medical comorbidities.

**Methods:**

Hospital admissions of adults with a primary diagnosis of heart failure and a history of third-degree heart block during the period between 2017 and 2022 were selected from the Healthcare Cost and Utilization Project National Inpatient Sample. The primary outcome was all-cause mortality; secondary outcomes were hospital length of stay and total hospital charges. Propensity matching was performed to account for differences between the two sample populations and reduce selection bias. Mortality was analyzed using logistic regression; the secondary outcomes were analyzed by using linear regression.

**Results:**

There were 22,900 prepandemic (2017–2019) hospitalizations of patients with heart failure and third-degree heart block and 37,530 hospitalizations during the pandemic (2020–2022). There was no associated difference in all-cause mortality (*p* = 0.36), length of hospital stay (*p* = 0.066), or total hospital charges (*p* = 0.65) during the pandemic. An increased odds of in-hospital mortality was associated with the presence of chronic pulmonary disease [odds ratio (OR): 1.79, 95% confidence interval (CI): 1.07–3.01, *p* = 0.027], valvular disease (OR: 1.63, 95% CI: 1.01–2.63, *p* = 0.046), uncomplicated diabetes (OR: 1.89, 95% CI: 1.02–3.51, *p* = 0.042), liver disease (OR: 3.22, 95% CI: 1.79–5.79, *p* < 0.001), and coagulopathy (OR: 1.97, 95% CI: 1.18–3.30, *p* = 0.010).

**Conclusion:**

There was no change in all-cause mortality length of stay or total charges of hospitalized patients with heart failure and a history of third-degree heart block during the COVID-19 pandemic as compared to before the pandemic. Certain comorbidities, however, were associated with higher mortality in this population.

## Introduction

Heart failure is one of the leading causes of hospitalization across the United States and a major cause of mortality and nationwide healthcare spending. Its prevalence in the country is projected to increase by approximately 50% from 2012 to 2030 (1). A major portion of healthcare costs due to heart failure is directly associated with hospitalizations, and recent literature has estimated an annual cost of $18 billion for heart failure hospitalizations in the United States (2). Along with significant economic burden, heart failure presents as a major cause of mortality, with studies showing an estimated 35% 1-year postdischarge mortality rate for adults aged 65 and older who are hospitalized for heart failure in the country (3). Third-degree heart block impairs left ventricular compliance and reduces cardiac output, linking it to the development of heart failure (4). Third-degree heart block presents significant morbidity and mortality for patients, as they experience risk for severe bradycardia and hemodynamic instability (5). This condition thus requires patients to have pacing with a permanent pacemaker.

The COVID-19 pandemic presented significant challenges for the national healthcare system, having a profound impact on healthcare outcomes and utilization. A large study using data from Medicare hospital patients found a significant increase in the rate of adverse events such as medication errors and hospital-acquired infections associated with a higher COVID-19 hospital burden (6).

Heart failure admissions are particularly affected by COVID-19. Several studies have reported that patients with heart failure during the COVID-19 pandemic experienced increased in-hospital mortality, increased length of hospital stay, and higher total hospital costs (7, 8). However, no research has been done to assess similar outcomes during the COVID-19 pandemic in hospitalized heart failure patients with a history of third-degree heart block. The goal of this study will be to better understand whether this particular high-risk group of patients also experienced worse outcomes during the COVID-19 pandemic.

## Methods

### National Inpatient Sample database

The National Inpatient Sample (NIS) was used to collect data for admissions between 2017 and 2022. As the largest publicly accessible all-payer inpatient healthcare database, the NIS estimates approximately 35 million hospitalizations nationally (9). NIS data elements include hospital characteristics, patient demographic characteristics, expected payment source, total charges, discharge status, length of stay, and severity and comorbidity measures (9). These data elements are grouped by admission for each hospital stay rather than for each patient. Since 2016, the NIS has used the International Classification of Diseases, Tenth Revision, Clinical Modification/Procedure Coding System (ICD-10-CM/PCS) (9). These deidentified data for this study did not require approval from an institutional review board.

### Patient population

**Figure 1:**
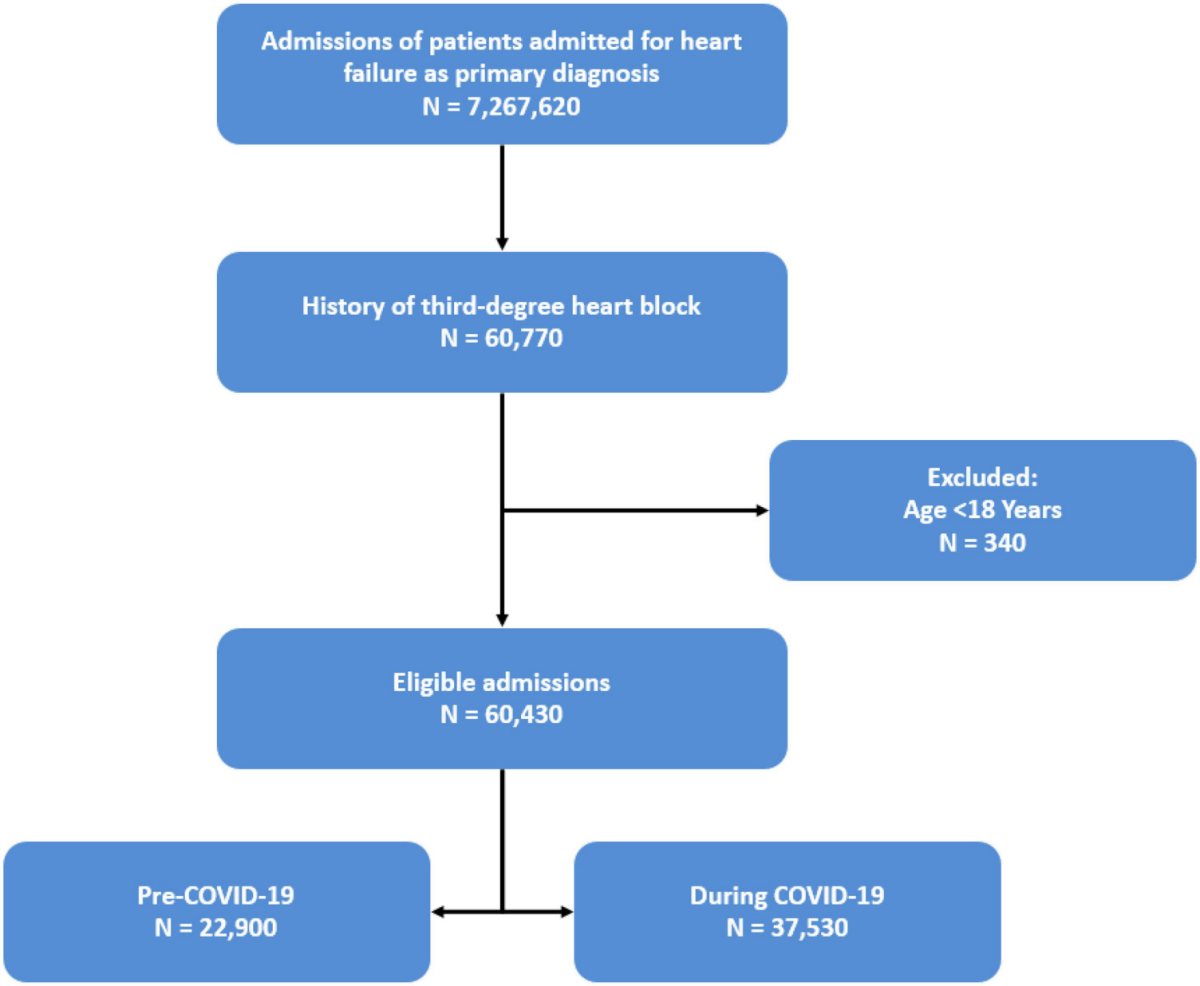
Patient sample selection.

Our initial patient population includes patients admitted to the hospital between 2017 and 2022 with a primary diagnosis of heart failure and a history of third-degree heart block. These patients were selected using the ICD-10 codes 150.x, 109.81, I11.0, I13.0, and I13.2 for heart failure and 144.2 for third-degree heart block. Patients were excluded if their age was less than 18 years old (Figure 1). We defined the period before the COVID-19 pandemic as 2017–2019 and the period during the COVID-19 pandemic as 2020–2022. Baseline clinical characteristics and differences between these two populations are included in Table 1. To account for these differences and reduce selection bias, propensity score matching was performed on whether the admissions were before or during the COVID-19 pandemic.

**Table 1:** Baseline demographic characteristics.

Variable	Overall *N* = 60,430^a^	During COVID-19 pandemic *N* = 37,530^a^	Not during COVID-19 pandemic *N* = 22,900^a^	*p*-Value^b^
Age, years	76.2 ± 0.1	76.6 ± 0.2	75.6 ± 0.2	0.002
Biological sex				0.126
Female	25,440 (42%)	16,005 (43%)	9,435 (41%)	
Male	34,990 (58%)	21,525 (57%)	13,465 (59%)	
Race				0.157
White	43,480 (74%)	27,365 (75%)	16,115 (73%)	
Black	8,555 (15%)	5,190 (14%)	3,365 (15%)	
Hispanic	4,425 (7.6%)	2,665 (7.3%)	1,760 (8.0%)	
Asian or Pacific Islander	1,310 (2.2%)	755 (2.1%)	555 (2.5%)	
Native American	340 (0.6%)	220 (0.6%)	120 (0.5%)	
Other	340 (0.6%)	245 (0.7%)	95 (0.4%)	
Insurance status				–
Medicare	48,745 (84%)	30,310 (84%)	18,435 (83%)	
Medicaid	3,405 (5.8%)	2,090 (5.8%)	1,315 (6.0%)	
Private insurance	6,180 (11%)	3,830 (11%)	2,350 (11%)	
Other	0 (0%)	0 (0%)	0 (0%)	
Hospital teaching status				0.096
Urban teaching	47,860 (79%)	30,010 (80%)	17,850 (78%)	
Rural	3,270 (5.4%)	2,035 (5.4%)	1,235 (5.4%)	
Urban non-teaching	9,300 (15%)	5,485 (15%)	3,815 (17%)	
Hospital region				0.491
Northeast	12,660 (21%)	8,105 (22%)	4,555 (20%)	
Midwest or north central	14,015 (23%)	8,800 (23%)	5,215 (23%)	
South	22,385 (37%)	13,700 (37%)	8,685 (38%)	
West	11,370 (19%)	6,925 (18%)	4,445 (19%)	
Median household income in zip code				0.001
0–25th percentile	14,900 (25%)	8,735 (24%)	6,165 (27%)	
26th to 50th percentile	15,270 (26%)	9,720 (26%)	5,550 (25%)	
51st to 75th percentile	15,185 (25%)	9,525 (26%)	5,660 (25%)	
76th to 100th percentile	14,285 (24%)	9,140 (25%)	5,145 (23%)	
Hospital bed size				0.162
Small	11,360 (19%)	7,370 (20%)	3,990 (17%)	
Medium	16,230 (27%)	10,025 (27%)	6,205 (27%)	
Large	32,840 (54%)	20,135 (54%)	12,705 (55%)	
Weekend admission				0.258
Weekday	47,040 (78%)	29,340 (78%)	17,700 (77%)	
Weekend	13,390 (22%)	8,190 (22%)	5,200 (23%)	
Elective admission				<0.001
Non-elective	57,240 (95%)	35,840 (96%)	21,400 (94%)	
Elective	3,145 (5.2%)	1,660 (4.4%)	1,485 (6.5%)	

a Mean ± SE; *n* (%).

b Design-based Kruskal–Wallis test; Pearson's *χ*^2^: Rao and Scott adjustment.

### Study variables and outcomes

The primary outcome for our study was in-hospital all-cause mortality. The secondary outcomes were length of hospital stay and total hospital charges. These outcomes were compared between our patient populations before the COVID-19 pandemic and during the COVID-19 pandemic, defined as the periods of 2017–2019 and 2020–2022, respectively.

The other variables for this study were patient demographics such as age, sex, race, and insurance status. We included hospital information such as region, teaching status, bed size, and median household income in zip code. We also included comorbidities such as the Elixhauser sum, a quantitative score that indicates a patient's overall comorbidity burden based on weighted scores of 38 common chronic diseases. The other comorbidity variables included for our dataset were chronic pulmonary disease, valvular disease, peripheral vascular disease, uncomplicated hypertension, diabetes, hypothyroidism, renal disease, liver disease, coagulopathy, obesity, weight loss, fluid and electrolyte disorders, anemia, alcohol abuse, dyslipidemia, smoking, atrial fibrillation, previous cerebrovascular disease (CVD), previous myocardial infarction (MI), previous coronary artery bypass grafting (CABG), and previous percutaneous coronary intervention (PCI). All of these variables were obtained from the NIS database using ICD-10 codes.

### Data analysis

All data analyses were conducted using R version 4.4.2. The data were weighted using survey weights provided by the NIS. In particular, the DISCWT variable was used as the discharge weight, HOSP_NIS as the primary sampling unit (i.e., hospital identifier), and NIS_STRATUM as the stratum variable. To minimize selection bias, we used propensity score stratification. Propensity scores were estimated by a logistic regression model using the COVID-19 pandemic status as the outcome. All covariates, including clinical comorbidities, patient demographics, and hospital characteristics, were controlled. The propensity score was subclassified into six groups using the MatchIt package in R. The quality of the stratification was evaluated by comparing standardized mean differences and variance ratios for covariates used to estimate the propensity score. The decision to retain non-linear forms was determined using the likelihood ratio test. Categorical variables were reported as counts with percentages and compared using Pearson's *χ*^2^ tests with Rao and Scott adjustment. Continuous variables were reported as means with standard error (SE) and compared using design-based Kruskal–Wallis tests. This non-parametric approach does not assume normality, and therefore, additional normality testing was not required for baseline comparisons. For comparisons limited to two periods such as pre- and during COVID-19, the design-based Kruskal–Wallis test is equivalent to a weighted Wilcoxon rank-sum test.

In-hospital mortality was the primary outcome, which was analyzed using logistic regression and reported as an odd ratio with a 95% confidence interval (CI; null value = 1). Our other outcomes were length of hospital stay and total hospital charges (adjusted for inflation). Both of these were analyzed using a linear regression model and reported as a mean difference in days spent in the hospital or total charges in dollars with 95% confidence intervals (null value = 0). All analyses were accounted for the NIS sampling design and were weighted to estimate national-level effects. A two-tailed *p* < 0.05 was defined to indicate statistical significance.

## Results

### Clinical characteristics

There were a total of 60,430 hospitalizations for patients with heart failure and third-degree heart block. Of these, 22,900 were hospitalized before the COVID-19 pandemic and 37,530 were hospitalized during the pandemic. The average age of the overall population studied was 76.2 ± 0.1 years. The average age of patients hospitalized before the pandemic was 75.6 ± 0.2 years as compared to the average age of those hospitalized during the pandemic, which was 76.6 ± 0.2 years (*p* = 0.002). Overall, sex, race, and insurance status inpatient admissions were predominantly 58% male, 74% white, and 84% with Medicare. The distribution of these variables did not differ significantly between patients hospitalized during the pandemic and those hospitalized before it (Table 1).

The majority of admissions occurred at urban teaching hospitals (79%), while only 5.4% of admissions occurred in rural hospitals. There was no statistically significant difference in admissions based on hospital teaching status before the COVID-19 pandemic and during the pandemic (*p* = 0.096). The regional distribution of the overall hospitalizations showed that 37% occurred in the South and only 19% occurred in the West. There was no significant difference in the distribution of hospital region between hospitalizations during the pandemic and those before it (*p* = 0.491). The median household income in the zip code of the hospital did show a statistically significant difference (*p* = 0.001) between the two groups, with a slightly higher proportion of patients in the lowest income quartile (27%) in the group of hospitalizations that did not occur during the pandemic compared with the group of hospitalizations that occurred during the pandemic (24%). Hospital bed size did not differ significantly between the two groups (*p* = 0.162), with the overall distribution being 54% large and 19% small. Most admissions were on weekdays (78%), with no significant difference between the two groups. Finally, there was a statistically significant difference in the proportion of elective admissions (*p* < 0.001), with 4.4% of the admissions being elective during the pandemic and 6.5% of the admissions being elective before the pandemic.

Table 2 describes differences in clinical attributes based on ICD-10 codes between patients admitted before the COVID-19 pandemic and those admitted during the pandemic. Complicated hypertension was more prevalent in hospitalizations during the pandemic (94% vs. 91%, *p* < 0.001). There was also a higher prevalence of complicated diabetes (40% vs. 36%, *p* < 0.001), obesity (23% vs. 21%, *p* = 0.017), dyslipidemia (64% vs. 58%, *p* < 0.001), and atrial fibrillation (58% vs. 54%, *p* < 0.001) during the pandemic. During the pandemic, 1.6% of hospitalizations had a positive COVID-19 status. However, during the pandemic, there was a lower proportion of hospitalizations with chronic pulmonary disease (33% vs. 35%, *p* = 0.020), uncomplicated hypertension (0.9% vs. 1.9%, *p* < 0.001), uncomplicated diabetes (7.6% vs. 9.8%, *p* < 0.001), smoking status (33% vs. 36%, *p* = 0.013), and history of CABG (14% vs. 16%, *p* = 0.015).

**Table 2:** Baseline clinical characteristics.

Variable	Overall *N* = 60,430^a^	During COVID-19 pandemic *N* = 37,530^a^	Not during COVID-19 pandemic *N* = 22,900^a^	*p*-Value^b^
Elixhauser sum	6.84 ± 0.02	6.89 ± 0.02	6.75 ± 0.03	<0.001
Chronic pulmonary disease	20,200 (33%)	12,240 (33%)	7,960 (35%)	0.020
Valvular disease	27,220 (45%)	17,015 (45%)	10,205 (45%)	0.435
Pulmonary circulation disease	16,750 (28%)	10,640 (28%)	6,110 (27%)	0.056
Peripheral vascular disease	12,845 (21%)	7,965 (21%)	4,880 (21%)	0.915
Uncomplicated hypertension	755 (1.2%)	325 (0.9%)	430 (1.9%)	<0.001
Paralysis	310 (0.5%)	175 (0.5%)	135 (0.6%)	0.354
Other neurological disorders	5,175 (8.6%)	3,410 (9.1%)	1,765 (7.7%)	0.009
Uncomplicated diabetes	5,105 (8.4%)	2,855 (7.6%)	2,250 (9.8%)	<0.001
Complicated diabetes	23,230 (38%)	15,005 (40%)	8,225 (36%)	<0.001
Hypothyroidism	12,605 (21%)	7,995 (21%)	4,610 (20%)	0.139
Renal failure and disease	34,960 (58%)	21,865 (58%)	13,095 (57%)	0.258
Liver disease	3,750 (6.2%)	2,425 (6.5%)	1,325 (5.8%)	0.139
Peptic ulcer	340 (0.6%)	205 (0.5%)	135 (0.6%)	0.757
Acquired immune deficiency syndrome	95 (0.2%)	65 (0.2%)	30 (0.1%)	0.569
Lymphoma	780 (1.3%)	475 (1.3%)	305 (1.3%)	0.755
Metastatic cancer	645 (1.1%)	375 (1.0%)	270 (1.2%)	0.359
Solid tumor without metastasis	1,680 (2.8%)	1,040 (2.8%)	640 (2.8%)	0.939
Rheumatoid arthritis	2,220 (3.7%)	1,330 (3.5%)	890 (3.9%)	0.337
Coagulopathy	6,575 (11%)	4,155 (11%)	2,420 (11%)	0.403
Obesity	13,700 (23%)	8,790 (23%)	4,910 (21%)	0.017
Weight loss	4,890 (8.1%)	2,945 (7.8%)	1,945 (8.5%)	0.231
Fluid and electrolyte disorders	25,450 (42%)	16,140 (43%)	9,310 (41%)	0.013
Chronic blood loss anemias	610 (1.0%)	365 (1.0%)	245 (1.1%)	0.604
Deficiency anemias	5,980 (9.9%)	3,920 (10%)	2,060 (9.0%)	0.011
Alcohol abuse	1,630 (2.7%)	985 (2.6%)	645 (2.8%)	0.529
Drug abuse	1,475 (2.4%)	975 (2.6%)	500 (2.2%)	0.164
Psychoses	375 (0.6%)	200 (0.5%)	175 (0.8%)	0.126
Depression	6,940 (11%)	4,395 (12%)	2,545 (11%)	0.337
Complicated hypertension	55,945 (93%)	35,105 (94%)	20,840 (91%)	<0.001
COVID-19	600 (1.0%)	600 (1.6%)	0 (0%)	<0.001
Dyslipidemia	37,210 (62%)	23,845 (64%)	13,365 (58%)	<0.001
Smoking	20,695 (34%)	12,515 (33%)	8,180 (36%)	0.013
Previous MI	8,870 (15%)	5,345 (14%)	3,525 (15%)	0.103
Previous CABG	9,030 (15%)	5,370 (14%)	3,660 (16%)	0.015
Previous PCI	7,590 (13%)	4,630 (12%)	2,960 (13%)	0.361
Previous CVD	7,570 (13%)	4,615 (12%)	2,955 (13%)	0.334
Atrial fibrillation	34,245 (57%)	21,920 (58%)	12,325 (54%)	<0.001

a Mean ± SE; *n* (%).

b Design-based Kruskal–Wallis test; Pearson's *χ*^2^: Rao and Scott adjustment.

There were several comorbidities for which there was no significant difference in prevalence between the two groups. These included valvular disease (45% overall), pulmonary circulation disease (28% overall), peripheral vascular disease (21% overall), coagulopathy (11% overall), weight loss (8.1% overall), previous MI (15% overall), previous PCI (13% overall), and previous CVD (13% overall).

### Mortality

Propensity-matched data for in-hospital mortality in our patient population are represented in Table 3 based on different clinical characteristics and their respective ICD-10 codes. The propensity-matched data indicate there was no significant increase in mortality for patients with a primary diagnosis of heart failure and a history of third-degree heart block during the COVID-19 pandemic, as compared to before the pandemic (*p* = 0.36). Similarly, admission year (*p* = 0.22) and biological sex (*p* = 0.32) did not show a significant association with in-hospital mortality.

**Table 3:** Propensity-matched in-hospital mortality.

Variable	OR	95% CI	*p*-Value
During COVID-19 pandemic	0.83	0.56, 1.23	0.36
Year	0.91	0.79, 1.06	0.22
Age, years	1.02	1.00, 1.03	0.009
Biological sex			
Female	–	–	
Male	1.12	0.89, 1.42	0.32
Race			
White	–	–	
Black	0.70	0.49, 1.02	0.062
Hispanic	0.87	0.56, 1.35	0.53
Asian or Pacific Islander	0.68	0.31, 1.51	0.35
Native American	0.57	0.08, 4.02	0.57
Other	0.46	0.06, 3.83	0.48
Insurance status			
Medicare	–	–	
Medicaid	1.49	0.88, 2.54	0.14
Private insurance	0.89	0.59, 1.33	0.56
Hospital teaching status			
Urban teaching	–	–	
Rural	1.00	0.58, 1.72	>0.99
Urban non-teaching	0.89	0.65, 1.22	0.47
Hospital region			
Northeast	–	–	
Midwest or north central	0.92	0.66, 1.28	0.62
South	0.87	0.64, 1.18	0.37
West	0.75	0.52, 1.06	0.10
Median household income in zip code			
0–25th percentile	–	–	
26th to 50th percentile	0.97	0.71, 1.35	0.88
51st to 75th percentile	1.03	0.75, 1.41	0.88
76th to 100th percentile	0.90	0.64, 1.28	0.56
Hospital bed size			
Small	–	–	
Medium	1.16	0.82, 1.63	0.41
Large	1.10	0.81, 1.50	0.55
Weekend admission			
Weekday	–	–	
Weekend	1.14	0.88, 1.48	0.32
Elective admission			
Non-elective	–	–	
Elective	0.77	0.44, 1.36	0.37
Elixhauser sum	0.71	0.46, 1.08	0.11
Chronic pulmonary disease	1.79	1.07, 3.01	0.027
Valvular disease	1.63	1.01, 2.63	0.046
Pulmonary circulation disease	1.32	0.81, 2.16	0.26
Peripheral vascular disease	1.09	0.64, 1.84	0.76
Uncomplicated hypertension	2.43	0.79, 7.54	0.12
Paralysis	5.44	1.98, 14.9	0.001
Other neurological disorders	4.47	2.71, 7.37	<0.001
Uncomplicated diabetes	1.89	1.02, 3.51	0.042
Complicated diabetes	1.50	0.89, 2.52	0.13
Hypothyroidism	1.73	1.07, 2.79	0.026
Renal failure and disease	1.63	0.98, 2.74	0.062
Liver disease	3.22	1.79, 5.79	<0.001
Peptic ulcer	0.34	0.09, 1.35	0.13
Acquired immune deficiency syndrome	0.00	0.00, 0.00	<0.001
Lymphoma	1.45	0.56, 3.74	0.45
Metastatic cancer	2.80	1.01, 7.75	0.048
Solid tumor without metastasis	0.95	0.37, 2.43	0.92
Rheumatoid arthritis	2.08	1.05, 4.10	0.035
Coagulopathy	1.97	1.18, 3.30	0.010
Obesity	1.29	0.76, 2.18	0.34
Weight loss	2.08	1.26, 3.45	0.004
Fluid and electrolyte disorders	3.90	2.32, 6.55	<0.001
Chronic blood loss anemias	0.74	0.18, 3.12	0.68
Deficiency anemias	0.91	0.51, 1.65	0.76
Alcohol abuse	0.45	0.16, 1.27	0.13
Drug abuse	2.14	0.89, 5.14	0.090
Psychoses	0.00	0.00, 0.01	<0.001
Depression	1.09	0.63, 1.90	0.75
COVID-19	1.10	0.45, 2.66	0.83
Dyslipidemia	0.80	0.63, 1.00	0.055
Smoking	0.63	0.48, 0.83	0.001
Previous MI	1.08	0.77, 1.51	0.67
Previous CABG	1.00	0.71, 1.42	0.98
Previous PCI	0.83	0.57, 1.20	0.32
Previous CVD	0.67	0.45, 0.99	0.045
Atrial fibrillation	0.81	0.65, 1.01	0.061

OR, odds ratio; CI, confidence interval.

Increasing age was shown to be associated with a statistically significant increase in in-hospital mortality [odds ratio (OR): 1.02, 95% CI: 1.00–1.03, *p* = 0.009]. There were several other comorbidities that were associated with a higher mortality, including weight loss (OR: 2.08, 95% CI: 1.26–3.45, *p* = 0.004), coagulopathy (OR: 1.97, 95% CI: 1.18–3.30, *p* = 0.010), chronic pulmonary disease (OR: 1.79, 95% CI: 1.07–3.01, *p* = 0.027), liver disease (OR: 3.22, 95% CI: 1.79–5.79, *p* < 0.001), valvular disease (OR: 1.63, 95% CI: 1.01–2.63, *p* = 0.046), and uncomplicated diabetes (OR: 1.89, 95% CI: 1.02–3.51, *p* = 0.042). Smoking was associated with lower in-hospital mortality (OR: 0.63, 95% CI: 0.48–0.83, *p* = 0.001), as was having a history of cardiovascular disease (OR: 0.67, 95% CI: 0.45–0.99, *p* = 0.045).

Several other variables were not significantly associated with in-hospital mortality, such as race, insurance status, hospital characteristics, weekend admission, and elective admission status. There were also a number of comorbidities that were not associated with a difference in in-hospital mortality, including pulmonary circulation disease, peripheral vascular disease, uncomplicated hypertension, complicated diabetes, dyslipidemia, atrial fibrillation, previous MI, previous CABG, and previous PCI. Notably, COVID-19 infection itself was not a significant predictor of in-hospital mortality in this cohort (*p* = 0.83).

### Length of stay

Table 4 describes propensity-matched data for length of hospital stay in this population based on different clinical attributes. Admission during the COVID-19 pandemic was not associated with a difference in length of stay (*p* = 0.066) between patients with a primary diagnosis of heart failure and a history of third-degree heart block. Similarly, admission year (*p* = 0.13) and male biological sex (*p* = 0.20) were not associated with changes in length of stay.

**Table 4:** Propensity-matched hospital length of stay.

Variable	Mean difference	95% CI	*p*-Value
During COVID -19 pandemic	−0.63	−1.3, 0.04	0.066
Year	0.17	−0.05, 0.39	0.13
Age, years	−0.08	−0.11, −0.05	<0.001
Biological sex			
Female	—	—	
Male	0.23	−0.13, 0.60	0.20
Race			
White	—	—	
Black	−0.41	−0.95, 0.12	0.13
Hispanic	−0.19	−0.86, 0.49	0.59
Asian or Pacific Islander	1.4	−1.4, 4.2	0.32
Native American	−1.1	−2.3, 0.08	0.067
Other	0.18	−1.3, 1.7	0.81
Insurance status			
Medicare	—	—	
Medicaid	0.52	−1.3, 2.4	0.58
Private insurance	0.13	−0.51, 0.77	0.68
Hospital teaching status			
Urban teaching	—	—	
Rural	−1.7	−2.3, −1.2	<0.001
Urban non-teaching	−0.96	−1.3, −0.60	<0.001
Hospital region			
Northeast	—	—	
Midwest or north central	−1.3	−1.7, −0.87	<0.001
South	−0.50	−0.96, −0.03	0.035
West	−1.6	−2.1, −1.0	<0.001
Median household income in zip code			
0–25th percentile	—	—	
26th to 50th percentile	−0.08	−0.64, 0.48	0.78
51st to 75th percentile	−0.24	−0.72, 0.25	0.34
76th to 100th percentile	−0.21	−0.70, 0.28	0.40
Hospital bed size			
Small	—	—	
Medium	0.05	−0.41, 0.51	0.83
Large	1.4	0.88, 1.9	<0.001
Weekend admission			
Weekday	—	—	
Weekend	−0.19	−0.64, 0.27	0.41
Elective admission			
Non-elective	—	—	
Elective	0.68	−0.75, 2.1	0.35
Elixhauser sum	0.52	−0.19, 1.2	0.15
Chronic pulmonary disease	−0.59	−1.4, 0.28	0.18
Valvular disease	−0.60	−1.5, 0.25	0.17
Pulmonary circulation disease	0.24	−0.55, 1.0	0.54
Peripheral vascular disease	−0.39	−1.2, 0.43	0.35
Uncomplicated hypertension	−0.21	−1.7, 1.2	0.77
Paralysis	0.15	−2.5, 2.9	0.91
Other neurological disorders	1.3	0.30, 2.3	0.010
Uncomplicated diabetes	−0.63	−1.5, 0.22	0.14
Complicated diabetes	−0.13	−1.1, 0.79	0.78
Hypothyroidism	−0.43	−1.2, 0.34	0.27
Renal failure and disease	0.16	−0.55, 0.87	0.65
Liver disease	0.06	−0.91, 1.0	0.91
Peptic ulcer	−0.04	−1.7, 1.6	0.96
Acquired immune deficiency syndrome	0.63	−5.4, 6.6	0.84
Lymphoma	−1.4	−2.7, −0.02	0.047
Metastatic cancer	−1.7	−3.0, −0.33	0.015
Solid tumor without metastasis	0.35	−0.88, 1.6	0.58
Rheumatoid arthritis	−0.43	−1.5, 0.60	0.41
Coagulopathy	1.4	0.35, 2.4	0.009
Obesity	−0.38	−1.3, 0.53	0.41
Weight loss	2.7	1.6, 3.8	<0.001
Fluid and electrolyte disorders	2.0	1.4, 2.7	<0.001
Chronic blood loss anemias	−0.03	−1.5, 1.4	0.96
Deficiency anemias	0.49	−0.32, 1.3	0.23
Alcohol abuse	−1.5	−3.0, 0.04	0.056
Drug abuse	0.06	−3.2, 3.3	0.97
Psychoses	−4.6	−6.1, −3.1	<0.001
Depression	0.09	−0.94, 1.1	0.86
COVID-19	0.71	−0.72, 2.1	0.33
Dyslipidemia	−0.99	−1.4, −0.60	<0.001
Smoking	−0.75	−1.1, −0.41	<0.001
Previous MI	−0.57	−0.97, −0.18	0.005
Previous CABG	−0.41	−0.76, −0.06	0.022
Previous PCI	−0.01	−0.68, 0.65	0.97
Previous CVD	−0.48	−0.80, −0.16	0.003
Atrial fibrillation	0.71	0.35, 1.1	<0.001

CI, confidence interval.

Increasing age was associated with a shorter hospital stay (mean difference = −0.08 days, 95% CI: −0.11 to −0.05, *p* < 0.001). There was no difference in length of stay in admissions between individuals who identified as Black, Hispanic, Asian or Pacific Islander, Native American, or Other when compared with individuals who identified as white (*p*-values = 0.13, 0.59, 0.32, 0.067, 0.81, respectively) With regard to hospital teaching status, patients admitted to rural (mean difference = −1.7 days, 95% CI: −2.3 to −1.2, *p* < 0.001) or urban non-teaching hospitals (mean difference = −0.96 days, 95% CI: −1.3 to −0.60, *p* < 0.001) had shorter stays compared with urban teaching hospitals. Compared with the Northeast, patients admitted in the Midwest/North Central (mean difference = −1.3, 95% CI: −1.7 to −0.87, *p* < 0.001) and South (mean difference = −0.50 days, 95% CI: −0.96 to −0.03, *p* = 0.035), and West (mean difference = −1.6 days, 95% CI: −2.1 to −1.0, *p* < 0.001) also had significantly shorter hospitalizations. Only admission to large hospital beds was significantly associated with longer stays (mean difference = 1.4 days, 95% CI: 0.88–1.9, *p* < 0.001). Neither weekend admission (*p* = 0.41) nor elective admission (*p* = 0.35) was significantly associated with a difference in length of stay.

Among comorbidities, several of them were significantly associated with longer hospital stays. These included coagulopathy (mean difference = 1.4 days, 95% CI: 0.35–2.4, *p* = 0.009), weight loss (mean difference = 2.7 days, 95% CI: 1.6–3.8, *p* < 0.001), and atrial fibrillation (mean difference = 0.71 days, 95% CI: 0.35–1.1, *p* < 0.001). Conversely, several variables were associated with shorter stays, and these included dyslipidemia (mean difference = −0.99 days, 95% CI: −1.4 to −0.60, *p* < 0.001), smoking (mean difference = −0.75, 95% CI: −1.1 to −0.41, *p* < 0.001), previous myocardial infarction (mean difference = −0.57, 95% CI: −0.97 to −0.18, *p* = 0.005), previous CABG (mean difference = −0.41 days, 95% CI: −0.76 to −0.06, *p* = 0.022), and previous cerebrovascular disease (mean difference = −0.48 days, 95% CI: −0.80 to −0.16, *p* = 0.003). Other chronic conditions, including uncomplicated hypertension (*p* = 0.77) and obesity (*p* = 0.82), were not significantly associated with a difference in hospital length of stay. Notably, COVID-19 infection (*p* = 0.33) and Elixhauser sum (*p* = 0.15) were also not significantly associated with a difference in hospital length of stay.

### Total hospital charges

Propensity-matched data for total hospital charges adjusted for inflation based on different clinical characteristics in this patient population are represented in Table 5. Admission during the COVID-19 pandemic compared with before the COVID-19 pandemic was not significantly associated with a difference in total hospital charges (*p* = 0.65), nor was the year of admission (*p* = 0.41). Increasing age was significantly associated with reduced charges (mean difference = −$2,667, 95% CI: −$3,307 to −$2,027, *p* < 0.001). Male sex was associated with higher charges compared with female sex (mean difference = $12,044, 95% CI: $3,673–$20,415, *p* = 0.005). Black patients (mean difference = −$22,275, 95% CI: −$36,968 to −$7,583, *p* = 0.003) and Native American patients (mean difference = −$45,985, 95% CI: −$70,166 to −$21,803, *p* < 0.001) were associated with significantly lower charges compared with white patients. The charges were not significantly different for Hispanic, Asian or Pacific Islander, or other racial groups. Insurance status such as Medicaid (*p* = 0.62) did not show a significant difference in total hospital charges. Rural (mean difference = −$63,766, 95% CI: −$76,268 to −$51,263, *p* < 0.001) and urban non-teaching hospitals (mean difference = −$19,942, 95% CI: −$29,662 to −$10,222, *p* < 0.001) were associated with lower charges compared with urban teaching hospitals. Hospital charges were also significantly different depending on the region of the hospital, with the Midwest/North Central (mean difference = −$53,243, 95% CI: −$67,585 to −$38,900, *p* < 0.001), South (mean difference = −$26,031, 95% CI: −$41,250 to −$10,812, *p* < 0.001), and West (mean difference = −$22,256, 95% CI: −$38,947 to −$5,565, *p* = 0.009) all associated with lower charges compared with the Northeast. The median household income of the zip code in which the hospital was located, for example 26th–50th percentile (*p* = 0.85), did not show any association with a difference in total hospital charges. Large bed size hospitals were also associated with significantly higher charges compared with small bed size hospitals (mean difference = $41,354, 95% CI: $30,358 to $52,350, *p* < 0.001), while medium bed size hospitals did not have a significant difference in hospital charges. Elective admissions were associated with significantly higher charges compared with non-elective admissions (mean difference = $72,381, 95% CI: $45,268 to $99,494, *p* < 0.001), whereas weekend admission had no significant difference compared with weekday admissions (*p* = 0.69).

**Table 5:** Propensity-matched total hospital charges.

Variable	Mean difference in hospital total charges	95% CI	*p*-Value
During COVID-19 pandemic	−4,491	−23,778, 14,795	0.65
Year	−2,450	−8,338, 3,439	0.41
Age, years	−2,667	−3,307, −2,027	<0.001
Biological sex			
Female	–	–	
Male	12,044	3,673, 20,415	0.005
Race			
White	–	–	
Black	−22,275	−36,968, −7,583	0.003
Hispanic	3,389	−14,540, 21,319	0.71
Asian or Pacific Islander	9,921	−19,973, 39,816	0.52
Native American	−45,985	−70,166, −21,803	<0.001
Other	49,431	−11,242, 110,103	0.11
Insurance status			
Medicare	–	–	
Medicaid	−8,365	−41,571, 24,841	0.62
Private insurance	7,785	−8,613, 24,183	0.35
Hospital teaching status			
Urban teaching	–	–	
Rural	−63,766	−76,268, −51,263	<0.001
Urban non-teaching	−19,942	−29,662, −10,222	<0.001
Hospital region			
Northeast	–	–	
Midwest or north central	−53,243	−67,585, −38,900	<0.001
South	−26,031	−41,250, −10,812	<0.001
West	−22,256	−38,947, −5,565	0.009
Median household income in zip code			
0–25th percentile	–	–	
26th to 50th percentile	1,476	−13,876, 16,828	0.85
51st to 75th percentile	−8,313	−22,220, 5,593	0.24
76th to 100th percentile	−6,574	−20,662, 7,515	0.36
Hospital bed size			
Small	–	–	
Medium	1,177	−9,358, 11,713	0.83
Large	41,354	30,358, 52,350	<0.001
Weekend admission			
Weekday	–	–	
Weekend	2,223	−8,522, 12,968	0.69
Elective admission			
Non-elective	–	–	
Elective	72,381	45,268, 99,494	<0.001
Elixhauser sum	19,225	−901, 39,350	0.061
Chronic pulmonary disease	−33,002	−55,121, −10,883	0.003
Valvular disease	−14,704	−38,255, 8,847	0.22
Pulmonary circulation disease	−3,215	−24,655, 18,226	0.77
Peripheral vascular disease	−30,023	−53,255, −6,792	0.011
Uncomplicated hypertension	−16,487	−58,778, 25,803	0.44
Paralysis	39,751	−36,136, 115,637	0.30
Other neurological disorders	−14,166	−40,623, 12,292	0.29
Uncomplicated diabetes	1,595	−23,939, 27,130	0.90
Complicated diabetes	2,676	−18,930, 24,281	0.81
Hypothyroidism	−24,814	−45,547, −4,081	0.019
Renal failure and disease	−17,854	−40,535, 4,826	0.12
Liver disease	−21,780	−55,023, 11,463	0.20
Peptic ulcer	−1,407	−45,250, 42,435	0.95
Acquired immune deficiency syndrome	43,172	−127,882, 214,226	0.62
Lymphoma	−22,602	−57,398, 12,193	0.20
Metastatic cancer	−34,415	−79,669, 10,840	0.14
Solid tumor without metastasis	6,745	−34,385, 47,875	0.75
Rheumatoid arthritis	1,214	−35,053, 37,482	0.95
Coagulopathy	39,881	11,373, 68,388	0.006
Obesity	−33,120	−58,376, −7,865	0.010
Weight loss	57,814	29,421, 86,207	<0.001
Fluid and electrolyte disorders	15,731	−5,748, 37,211	0.15
Chronic blood loss anemias	−50,551	−78,980, −22,122	<0.001
Deficiency anemias	−45,589	−68,843, −22,335	<0.001
Alcohol abuse	−53,032	−87,011, −19,054	0.002
Drug abuse	−24,076	−82,731, 34,579	0.42
Psychoses	−106,326	−157,725, −54,928	<0.001
Depression	−16,809	−37,425, 3,807	0.11
COVID-19	−12,699	−44,111, 18,712	0.43
Dyslipidemia	−22,684	−33,410, −11,959	<0.001
Smoking	−7,177	−16,537, 2,184	0.13
Previous MI	831	−10,308, 11,970	0.88
Previous CABG	−11,059	−20,390, −1,728	0.020
Previous PCI	−4,223	−13,720, 5,274	0.38
Previous CVD	−9,573	−18,101, −1,046	0.028
Atrial fibrillation	3,549	−5,589, 12,688	0.45

CI, confidence interval.

Comorbidities such as weight loss (mean difference = $57,814, 95% CI: $29,421 to $86,207, *p* < 0.001) and coagulopathy (mean difference = $39,881, 95% CI: $11,373 to $68,388, *p* = 0.006) were associated with higher total hospital charges. Conversely, there were many comorbidities that were associated with decreased total hospital charges. These included chronic pulmonary disease (mean difference = −$33,002, 95% CI: −$55,121 to −$10,883, *p* = 0.003), obesity (mean difference = −$33,120, 95% CI: −$58,376 to −$7,865, *p* = 0.010), peripheral vascular disease (mean difference = −$30,023, 95% CI: −$53,255 to −$6,792, *p* = 0.011), dyslipidemia (mean difference = −$22,684, 95% CI: −$33,410 to −$11,959, *p* < 0.001), previous CABG (mean difference = −$11,059, 95% CI: −$20,390 to −$1,728, *p* = 0.020), and previous CVD (mean difference = −$9,573, 95% CI: −$18,101 to −$1,046, *p* = 0.028).

COVID-19 infection was also not significantly associated with a difference in total hospital charges (*p* = 0.43). Other comorbidities that did not have a significant difference in total hospital charges included valvular disease (*p* = 0.22), pulmonary circulation disorders *p* = 0.77), uncomplicated hypertension (*p* = 0.44), uncomplicated diabetes (*p* = 0.90), complicated diabetes (*p* = 0.81), smoking: (*p* = 0.13), previous MI (*p* = 0.88), previous PCI (*p* = 0.38), and atrial fibrillation (*p* = 0.45).

## Discussion

Despite the strain placed on the national healthcare system by the COVID-19 pandemic, our study found that there was no significant difference in in-hospital mortality before and during the COVID-19 pandemic among patients admitted with a primary diagnosis of heart failure and a history of third-degree heart block. There was also no significant difference in the length of hospital stay or total hospital charges adjusted for inflation for this same group. However, certain comorbidities among this population were associated with differences in these hospital outcomes.

We observed a decrease in the proportion of hospitalizations with chronic pulmonary disease during the COVID-19 pandemic. Similar reductions in admissions for chronic obstructive pulmonary disease (COPD) exacerbations have been reported in other studies, as evidenced by the systematic review and meta-analysis by Alqahtani et al. (10). The reduction in hospitalizations is likely multifactorial. Public health measures such as mask wearing, social distancing, and improved hand hygiene may have limited viral triggers of exacerbations (9). In addition, reductions in air pollution during lockdowns may have lessened environmental contributors to respiratory morbidity (11). The expansion of telemedicine and the prioritization of hospital resources for more severe disease could also have contributed to fewer inpatient admissions for chronic pulmonary conditions (12, 13).

Although our findings suggest no significant difference in in-hospital outcomes for this high-risk group, an important limitation is that the NIS database does not contain data relating to procedural details such as pacemaker implantation rates or potential delays in device therapy. This limitation is particularly relevant given that third-degree heart block almost universally requires permanent pacing. In a cohort of patients with heart failure, Lyons et al. reported that 57% met guideline-based indications for an intracardiac device (ICD) or cardiac resynchronization therapy (CRT), with yearly adherence rates of 59%–68% for ICDs and 66%–81% for CRT implantation among these eligible patients (14). Given that our study population also had third-degree heart block, it is likely that a substantial proportion would have met device indications. However, the NIS database cannot provide this information, and the lack of published data specifically addressing device eligibility in patients with both heart failure and third-degree heart block makes it challenging to estimate the true proportion or to assess the potential impact on outcomes.

Existing literature has shown that access to device implantation was affected during the COVID-19 pandemic. For example, Migliore et al. reported a significant decrease in urgent pacemaker implantations in the Veneto region of Italy during the early COVID-19 outbreak, raising concerns that delays in treatment for atrioventricular block may have contributed to adverse outcomes in that setting (15). While our study cannot determine whether similar disruptions occurred in the United States, the absence of differences in mortality, length of stay, or hospital charges in our cohort may indicate that US hospitals were able to preserve access to essential device-based therapies despite pandemic-related system strain. Future studies should examine pacemaker implantation rates in this population to clarify whether access to device therapy was maintained during the pandemic.

Studies using the NIS database have shown that the COVID-19 pandemic was associated with increased in-hospital mortality, length of hospital stay, and total hospital charges for patients admitted to the hospital with heart failure (7). For example, one study found that in-hospital mortality for patients with heart failure increased from 2.4% to 2.8% in 2020 as compared to before the pandemic in 2019 (7). To our knowledge, there is no study that directly assesses the in-hospital mortality of admissions of heart failure with third-degree heart block during the COVID-19 pandemic. However, one study found that the presence of third-degree heart block was an independent predictor of in-hospital mortality for admissions of ST-elevation myocardial infarction treated with PCI (16). Our study demonstrates a unique and unexpected lack of an increase in mortality for this high-risk group of patients. One study investigating outcomes for patients with heart failure during the COVID-19 pandemic found that there was a small increase in the average length of stay from 5.4 to 5.62 days as well as a large increase in the average hospital charges from $57,850 to $63,936 between the years 2019 and 2020 for patients with heart failure (7). Admissions of heart failure with third-degree heart block did not show such changes in length of hospital stay or total hospital charges.

Although there was no change in mortality for this patient population during the pandemic, certain comorbidities were associated with significant differences in mortality. Some of these comorbidities, including chronic pulmonary disease and uncomplicated diabetes, have been shown in other studies to increase the odds of mortality in patients with heart failure (17, 18). In addition, recent literature suggests that the presence of chronic liver disease and coagulopathy also leads to worse outcomes for patients with heart failure (19, 20). Our findings for hospital mortality in heart failure patients with third-degree heart block and these additional conditions appear to be consistent with the literature on heart failure.

Interestingly, we found several comorbidities to be associated with a decrease in the odds of hospital mortality for this patient population. These include a history of smoking and a history of cerebrovascular disease. The presence of smoking and cerebrovascular disease in patients with heart failure has been found in another study to significantly increase the odds of mortality, which is disagreement with our findings in this study (21). Our unexpected findings are most likely attributable to residual confounding factors that could not be obtained from the NIS database. Such confounders may include, among others, the duration of smoking history, the number of cigarettes smoked per day, and the severity of cerebrovascular disease. In addition, patients with significant smoking history or established cardiovascular or cerebrovascular disease may have been more likely to be deemed poor candidates for surgical intervention due to limited functional capacity, such as inability to achieve adequate metabolic equivalents, or due to clinician or surgeon discretion. As a result, there may have been a disproportionate use of device-based therapies in these patients, making unmeasured device implantation a potentially important residual confounder in this analysis.

Another potential contributing factor is survivor bias, whereby patients with comorbidities such as smoking-related disease or prior cerebrovascular events may represent a subgroup that survived long enough to be hospitalized. These patients may have possessed underlying biologic or genetic characteristics, as well as environmental factors such as more established outpatient care, that were associated with longer-term survival. Differential case mix may have also contributed, as patients with documented cardiovascular comorbidities may have been more likely to receive earlier recognition, closer inpatient monitoring, and more aggressive management during hospitalization. Finally, differences in clinical surveillance and management intensity, including a higher likelihood of telemetry monitoring, specialist consultation, or expedited escalation of care, could have favored patients with known cardiovascular comorbidities over those presenting without such histories. Together, these factors may have contributed to the paradoxical associations observed between these comorbidities and in-hospital mortality, underscoring the need for cautious interpretation. We encourage future researchers to investigate these risk factors in our population, particularly if they are able to control for these factors, especially the residual confounders previously mentioned.

The presence of peripheral vascular disease, renal disease, atrial fibrillation, previous MI or PCI, or previous CABG was not associated with any change in mortality within our patient population. Current literature suggests that the coexistence of renal disease, peripheral vascular disease, or documented atrial fibrillation is associated with worse outcomes in patients with heart failure, and specifically increased odds of in-hospital mortality (22–24). The use of PCI or CABG has previously been associated with changes in outcomes for patients with heart failure, and CABG has been shown to have a greater mortality benefit than PCI for revascularization in patients with complex heart failure (25). Altogether, the lack of association between these major comorbidities and in-hospital mortality may reflect differences in heart failure patients with third-degree heart block compared with the broader heart failure population. A review of the current literature reveals no studies that have investigated this difference, highlighting the need for further research on the unique characteristics of this patient population.

We also found no association with a longer length of hospital stay during the COVID-19 pandemic as compared to the prepandemic period from 2017 to 2019. Another study from a single urban hospital demonstrated that despite fewer overall heart failure admissions during the early part of the pandemic from March 2020 to October 2020, these patients experienced increased length of hospital stay and higher readmission rates (8). Again, this difference in findings may point to a difference in outcomes between the early and the later part of the pandemic for patients with heart failure, or it may highlight a key difference in outcomes between patients with heart failure in general and heart failure patients with third-degree heart block. The NIS does not report data regarding the readmission rate, but this could be an area of further exploration.

There was no significant change in hospital length of stay during the COVID-19 pandemic for patients admitted for heart failure and with a history of third-degree heart block. Many comorbidities were also not associated with any change in length of stay, including chronic pulmonary disease, peripheral vascular disease, diabetes, renal disease, liver disease, and previous history of PCI. However, atrial fibrillation and coagulopathy were both associated with an increased length of stay. Our results show that admissions with a history of atrial fibrillation had longer lengths of stay, in line with the current literature on admissions for heart failure (26). To our knowledge, the association between coagulopathy and heart failure on average hospital length of stay has not been studied, but one study found coagulopathy in patients admitted for COVID-19 to be a predictor of longer length of stay (27). We also found previous MI and previous CABG to be associated with decreased length of stay. However, the literature suggests that previous MI is not associated with any difference in length of stay for patients with heart failure (28).

Many comorbidities are not associated with any significant difference in total hospital charges, and these include valvular disease, diabetes, renal disease, liver disease, atrial fibrillation, previous MI, and previous PCI. We found coagulopathy to be associated with higher total hospital charges for heart failure patients with third-degree heart block. There does not seem to be any other current literature directly investigating this association, which provides an opportunity for further inquiry. Several comorbidities were associated with decreased hospital charges, including chronic pulmonary disease, peripheral vascular disease, and previous CABG. Current studies have shown the coexistence of pulmonary disease and peripheral vascular disease to be associated with higher total charges, while a history of CABG has been associated with lower charges (29).

The strengths of our study include the large population size from the NIS database. In addition, performing propensity score matching reduces selection bias arising from the original population groupings. One limitation of our study is its observational design using the NIS database, as no temporal claims or causal claims can be concluded. We also cannot account for errors related to incorrect billing codes or human mistakes in data input. Furthermore, we are unable to determine how many hospitalizations were readmissions of the same patient, as such data are not tracked in the NIS. Future studies should aim to expand on this lack of relevant clinical data wherever it may be possible, including but not limited to exploration of third-degree heart block vs. other types of heart block in these patients with heart failure to better understand the unique characteristics of hospitalizations in this specific population. The differences in the existing literature could be due to disease misclassification or residual confounding, as the NIS database cannot account for data on disease severity, medication use, or timing of diagnosis and treatments. Because of residual confounding variables that cannot be accounted for, these findings should be interpreted carefully and not seen as a protective effect.

## Conclusion

This study found that there was no significant change in in-hospital mortality, length of stay, or total hospital charges during the COVID-19 pandemic for patients admitted for heart failure with a history of third-degree heart block, suggesting that both the quality and the efficiency of hospital care was maintained for these high-risk patients during the pandemic.

## Data Availability

The original contributions presented in the study are included in the article/Supplementary Material, and further inquiries can be directed to the corresponding author.
